# QuickStats

**Published:** 2013-08-16

**Authors:** Hashini Khajuria, Shilpa Bengeri

**Figure:**
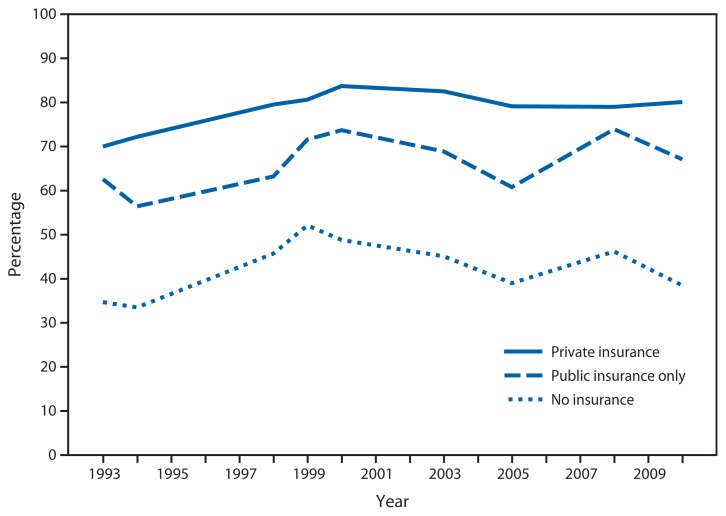
Percentage of Women Aged 50–64 Years Who Reported Receiving a Mammogram in the Past 2 Years, by Health Insurance Status^*†^ — National Health Interview Survey,^§^ United States, 1993–2010 ^*^ Questions concerning mammogram use have differed slightly over the years. Since 2000, respondents were asked for the date of their most recent mammogram; included are women who reported having had a mammogram in the past 2 years. Questions were administered as part of a cancer control supplement conducted in 1993, 1994, 1998, 1999, 2000, 2003, 2005, 2008, and 2010. ^†^ Health insurance status is coverage at the time of interview. Public insurance includes Medicaid, Medicare, Children’s Health Insurance Program, military, and other public assistance and government programs. Those with only Indian Health Service coverage are classified as uninsured. Because most women aged ≥65 years are covered by public insurance (Medicare), this figure presents data only for women aged 50–64 years. ^§^ Data are based on household interviews of a sample of the noninstitutionalized U.S. civilian population.

During 1993–2010, among women aged 50–64 years, insured women were more likely than uninsured women to report having a mammogram in the past 2 years. The percentage of privately insured women reporting a mammogram in the past 2 years rose from 70.0% in 1993 to 83.7% in 2000 and did not change significantly after 2000. Mammogram use among publicly insured and uninsured women aged 50–64 years varied during the period but was at approximately the same level in 1993 and 2010, and generally was lower than mammogram use among privately insured women. In 2010, 80.1% of women with private insurance, 67.1% of publicly insured women, and 38.5% of uninsured women aged 50–64 years had a mammogram in the past 2 years.

**Source:** National Health Interview Survey data. Available at http://www.cdc.gov/nchs/nhis/nhis_questionnaires.htm.

